# Metagenomic analysis provides functional insights into seasonal change of a non-cyanobacterial prokaryotic community in temperate coastal waters

**DOI:** 10.1371/journal.pone.0257862

**Published:** 2021-10-12

**Authors:** Kaoru Matsumoto, Tomoko Sakami, Tsuyoshi Watanabe, Yukiko Taniuchi, Akira Kuwata, Shigeho Kakehi, Tan Engkong, Yoji Igarashi, Shigeharu Kinoshita, Shuichi Asakawa, Masahira Hattori, Shugo Watabe, Yoshizumi Ishino, Takanori Kobayashi, Takashi Gojobori, Kazuho Ikeo

**Affiliations:** 1 Department of Genomics and Evolutionary Biology, National Institute of Genetics, Mishima, Shizuoka, Japan; 2 Tohoku National Fisheries Research Institute, Japan Fisheries Research and Education Agency, Shiogama, Miyagi, Japan; 3 Hokkaido National Fisheries Research Institute, Japan Fisheries Research and Education Agency, Kushiro, Hokkaido, Japan; 4 Department of Aquatic Bioscience, Graduate School of Agricultural and Life Sciences, The University of Tokyo, Bunkyo, Tokyo, Japan; 5 Graduate School of Advanced Science and Engineering, Waseda University, Shinjuku, Tokyo, Japan; 6 School of Marine Biosciences, Kitasato University, Sagamihara, Kanagawa, Japan; 7 Department of Bioscience and Biotechnology, Graduate School of Bioresource and Bioenvironmental Sciences, Kyushu University, Fukuoka, Fukuoka, Japan; 8 National Research Institute of Fisheries Science, Japan Fisheries Research and Education Agency, Yokohama, Kanagawa, Japan; Stazione Zoologica Anton Dohrn, ITALY

## Abstract

The taxonomic compositions of marine prokaryotic communities are known to follow seasonal cycles, but functional metagenomic insights into this seasonality is still limited. We analyzed a total of 22 metagenomes collected at 11 time points over a 14-month period from two sites in Sendai Bay, Japan to obtain seasonal snapshots of predicted functional profiles of the non-cyanobacterial prokaryotic community. Along with taxonomic composition, functional gene composition varied seasonally and was related to chlorophyll *a* concentration, water temperature, and salinity. Spring phytoplankton bloom stimulated increased abundances of putative genes that encode enzymes in amino acid metabolism pathways. Several groups of functional genes, including those related to signal transduction and cellular communication, increased in abundance during the mid- to post-bloom period, which seemed to be associated with a particle-attached lifestyle. Alternatively, genes in carbon metabolism pathways were generally more abundant in the low chlorophyll *a* period than the bloom period. These results indicate that changes in trophic condition associated with seasonal phytoplankton succession altered the community function of prokaryotes. Our findings on seasonal changes of predicted function provide fundamental information for future research on the mechanisms that shape marine microbial communities.

## Introduction

Prokaryotes play essential roles in oceanic biogeochemical cycles. Notably, heterotrophic prokaryotes process a large part of the organic matter produced by phytoplankton and are consumed by predators, thereby sustaining nutrient recycling [1–3]. 16S rRNA gene sequencing has been revealed spatial and temporal diversity of marine prokaryotic community structure in relation to the environmental conditions [4,5]. Seasonal cycle of surface prokaryotic community compositions has been well studies in various ocean locations, and are often associated with seasonal succession of the phytoplankton populations [5–10]. In temperate regions, the spring phytoplankton bloom is an important event that provides organic matter to heterotrophic prokaryotes, which results in increased prokaryotic production and biomass, and pronounced changes in community composition [11,12]. Some bacterial groups, such as flavobacteria and roseobacters, typically become dominant in the prokaryotic community during the phytoplankton bloom, whereas some other groups, such as pelagibacters (SAR11 clade), usually increase in abundance under low nutrient conditions during non-bloom periods [10,13]. Thus, one major factor of seasonal dynamics of prokaryotic community is considered to be variation in the suitable concentration and composition of nutrients among taxa due to diversification and specialization of their ability to utilize organic matters.

However, 16S rRNA gene sequencing itself only reveals phylogenetic diversity and does not provide insight into biological function. It is not easy to infer functional characteristics from marker gene sequences by referring to genome databases because the available genomes are limited and biological functions can be highly diverse, even among closely related taxa [14,15]. Therefore, shotgun metagenomics, which is random, non-targeted sequencing of all genomic DNA in an environment, has been applied to explore biological functions of microbial communities. Shotgun metagenomics assesses the “functional potential” of microbial communities based on the functional annotation of genes and their abundances, which refers to the kind of gene sequences present and their relative frequency in the metagenome [16]. For example, metagenomics has been used to investigate the functional diversity of marine microbial communities in different oceans [17], depths [18], and microhabitats [19,20]; moreover, metagenomics has been used to investigate the relationship between community structure and function [21–23]. However, only a few studies have examined seasonal differences in functional gene composition using metagenomes [23,24]. There are also few reports of functional estimates from marker gene composition [15]. Therefore, the functional aspect of seasonal cycles in prokaryotic communities still needs to be examined.

The aim of this study is to explore the functional aspect of the seasonal change of the prokaryotic community composition associated with the phytoplankton seasonal succession by using metagenomes. We analized time-series metagenomic datasets from Sendai Bay, Japan, where the seasonal succession of the phytoplankton community has been well described [25–27]. During the phytoplankton monitoring from March 2012 to April 2014 in Sendai Bay, they observed typical seasonal changes in a temperate ocean; chlorophyll *a* (Chl *a*) concentration, which indicates phytoplankton biomass, peaked in the spring (April and May), decreased in June and remained low during the summer, then gradually increased in the winter (around January) and peaked again in the following spring; spring bloom consisted of large diatoms, whereas cyanobacteria and picoeukaryote became dominant in the summer period [26]. The bacterial community in Sendai Bay was also investigated using 16S rRNA gene sequencing, and seasonal changes in the community composition was obserbed [28]. Marine microbial community studies often separate the microbes according to particle size by filtering seawater, and similary, the prokaryotic community from Sendai Bay was separated into mainly two fractions, 0.2–0.8μm fraction (mostly small, free-living prokaryotes) and 0.8–5μm fraction (relatively large prokaryotes and those attached to small particles, as well as picoeukaryotes). In this study we used a 0.8–5-μm fraction, since particle-attached microbes significantly contribute to degradation of phytoplankton-derived organic matter [13]. Due to the concern that the large increase of cyanobacterial and picoeukaryotes during summer period significantly affect the metagenomic comparison, we extracted sequences of prokaryotes excluding cyanobacteria and analyzed the taxonomic composition and functional gene composition. We show that the functional gene profile of the non-cyanobacterial prokaryotic community varied seasonally along with the taxonomic composition, and report the seasonal patterns of functional gene abundance that seemed to reflect changes in nutrient condition associated with the phytoplankton population.

## Materials and methods

### Sample collection

**Fig 1:**
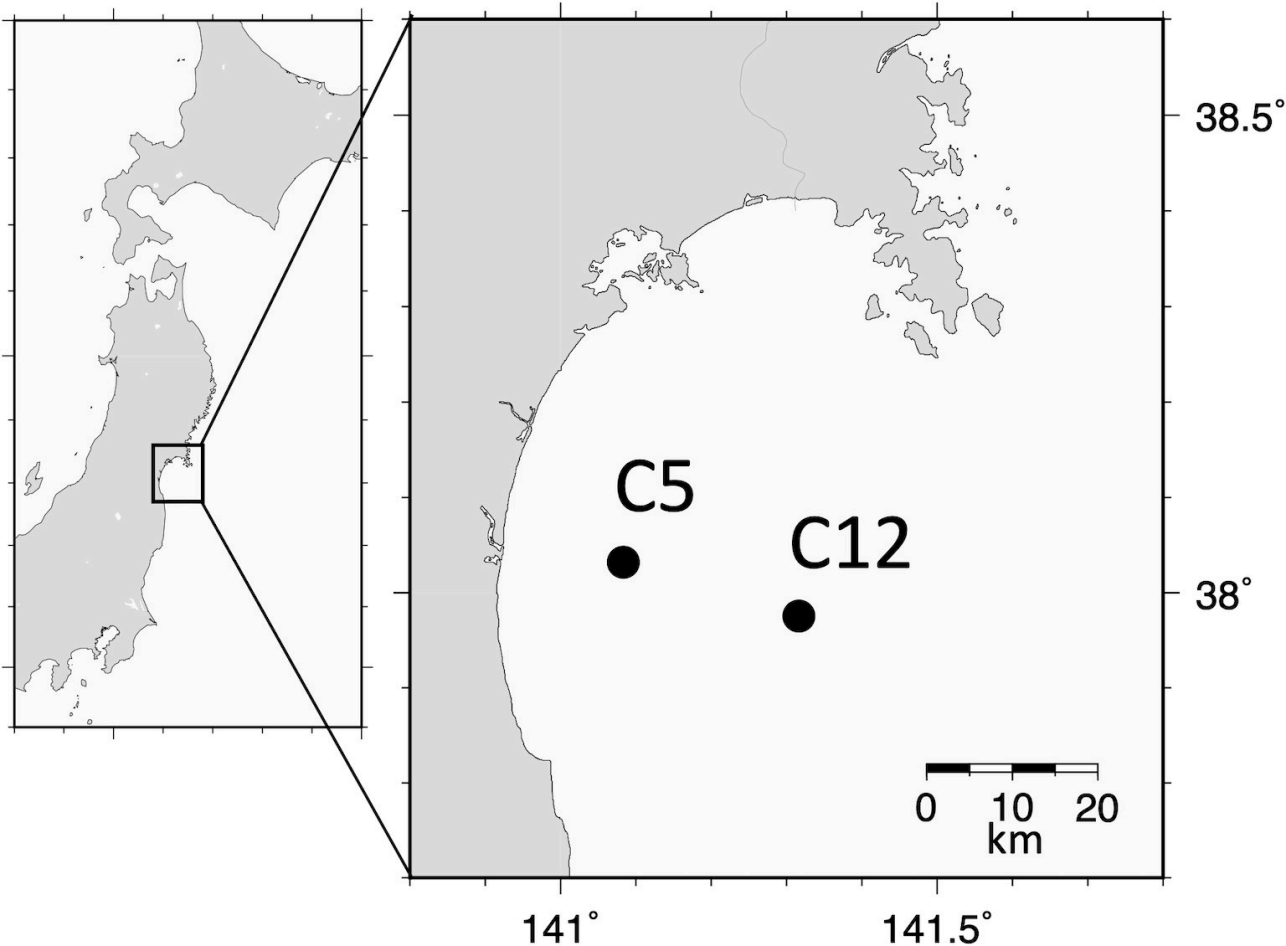
Map of Sendai Bay showing the sampling sites. These maps were prepared using the Generic Mapping Tools (https://www.generic-mapping-tools.org/).

The sampling location, Sendai Bay, is a simple semicircle shape with a wide opening toward the northwest Pacific Ocean. This bay covers an area of 2 000 km^2^ of shallow (mostly < 50 m) continental shelf, and some rivers flow into it. Our samplings were conducted under the permission by the Miyagi Fishery Co-operative. Approximately 7 L of surface water samples (approximately 1m below the sea surface) were collected at 11 time points between April 2012 and June 2013 from two sites off the Abukuma River, C5 (38.03°N, 141.08°E; bottom depth, 30 m) and C12 (37.98°N, 141.32°E; bottom depth, 60 m) (Fig 1). Large particles were removed immediately after collection using a 100-μm mesh plankton net. The seawater samples were sequentially filtered through a 20-μm mesh plankton net and nucleopore membrane filters with 5- and 0.8-μm pore sizes under positive pressure using a peristaltic pump. Each filter was stored at −80°C until DNA extraction. The environmental parameters, which included water temperature, salinity, and concentrations of Chl *a* and inorganic nutrients (nitrate, phosphate, and silicate), were measured in the surface and subsurface chlorophyll maxima, and were previously described [26,27]. Table 1 shows the sampling date and environmental variables in the surface layer.

**Table 1:** Sampling dates and environmental variables. The environmental variables were measured in surface water (≤ 1-m depth); only the values in square brackets were measured in the sub-surface chlorophyll *a* maximum (10- to 50-m depth).

Year	Date	Site	Temperature	Salinity	Chl *a*	NO_3_^-^	PO_4_^2-^	Si
			(°C)	(psu)	(μg/ml)	(μM)	(μM)	(μM)
2012	16 Apr	C5	7.3	33.1	2.5 [8.6]	0.2	0.1	1.2
	16 Apr	C12	6.9	33.0	5.7 [8.7]	0.2	0.1	1.2
	18 May	C5	13.2	31.4	2.6 [8.6]	0.2	0.1	1.8
	18 May	C12	13.0	31.4	1.7 [7.2]	0.1	0.1	1.7
	18 Jun	C5	17.8	31.4	0.7 [0.9]	0.2	0.1	6.7
	17 Jun	C12	17.5	31.3	0.7 [1.5]	0.3	0.1	5.4
	14 Jul	C5	19.2	31.4	1.9 [1.4]	0.2	0.1	4.5
	16 Jul	C12	18.3	32.9	0.3 [0.9]	0.2	0.1	1.9
	9 Aug	C5	23.6	32.0	0.4 [0.4]	0.2	0.0	3.6
	8 Aug	C12	21.2	33.3	0.3 [0.8]	0.2	0.1	2.2
	21 Sep	C5	24.4	33.5	0.6 [1.4]	0.3	0.1	1.9
	21 Sep	C12	22.9	33.8	0.9 [0.9]	0.3	0.1	3.9
	26 Nov	C5	14.8	33.5	1.9 [1.8]	1.0	0.4	9.4
	26 Nov	C12	16.0	33.8	1.1 [1.0]	1.3	0.2	7.4
2013	17 Jan	C5	10.3	34.1	2.7 [4.0]	2.1	0.2	4.5
	17 Jan	C12	9.5	34.0	1.6 [1.7]	2.3	0.3	5.5
	14 Mar	C5	8.8	34.1	7.6 [7.8]	1.0	0.2	3.6
	14 Mar	C12	8.3	34.0	3.3 [3.4]	2.9	0.4	6.4
	23 Apr	C5	8.9	33.1	3.2 [4.0]	0.1	0.1	1.7
	23 Apr	C12	8.1	33.0	8.4 [8.8]	0.1	0.1	5.9
	27 Jun	C5	20.0	32.5	0.5 [0.5]	0.1	0.1	3.2
	28 Jun	C12	16.6	33.1	0.8 [2.0]	0.1	0.1	2.2

### DNA extraction and metagenomic sequencing

Genomic DNA was extracted from a 0.8-μm pore size filter (0.8- to 5-μm size fraction) using a PowerWater DNA isolation kit (Mo Bio Laboratories, Solana Beach, CA), following the manufacturer’s instructions. DNA library preparation and sequencing were performed with kits from Life Technologies (Carlsbad, CA) following the manufacturer’s instructions. Library preparation and emulsion PCR were performed using the Ion Xpress Plus fragment library kit and Ion PGM Template OT2 400 kit. Sequencing was performed using an Ion Torrent PGM system (Life Technologies) with the Ion PGM Sequencing 400 kit and Ion 318 chip.

### Sequence data analysis

Raw read sequences were quality-controlled using a FASTX-Toolkit [29] as follows: the first 15 bases at the 5′ ends and the bases after the 230^th^ base at the 3′ ends were trimmed out; then, 3′ ends with a low quality score (< 20) were further trimmed out, and reads that were too short (< 90 bp) or too low in quality (less than 80% of the bases had quality scores ≥ 20) were removed. Reads with the same start position and nearly identical sequences were considered duplicates and also removed using cd-hit-454 [30] with the default settings (98% sequence identity). Protein-coding sequences were predicted with MetaGeneMark [31], and the longest peptide sequences predicted from each read that were longer than 30 amino acids were retained. To obtain taxonomic annotations, the predicted peptide sequences were subjected to blastp search against the NCBI-NR database (downloaded 15 April 2016) with an e-value threshold of 1e-10. The resulting files were analyzed by the weighted lowest common ancestor algorithm using MEGAN6 [32] (with the default parameters except ’min support percent: off’), which assigned the query sequences to the nodes of the NCBI taxonomy tree. The domain rank taxonomic composition (including viruses) of the predicted peptide sequences in each sample was obtained as the proportion of each domain in the total sequences assigned to any of the domain.

We extracted the predicted peptide sequences assigned to bacteria or archaea, except for those assigned to cyanobacteria, and analyzed their taxonomic and functional gene compositions. Statistical analyses and graphical representation were performed using R version 3 [33]; unless otherwise noted, the *vegan* package [34] was used.

Taxonomic composition of the prokaryotic sequences was generally based on phylum rank, but the abundantly detected classes (max abundance > 0.2), Flavobacteriia, Alphaproteobacteria, and Gammaproteobacteria, were distinguished from their parent phyla. Furthermore, given their abundance and different environmental preferences [10,13,35], Rhodobacterales, including Roseobacter, and Pelagibacterales, including SAR11, were distinguished from the parent class Alphaproteobacteria. The Bray–Curtis dissimilarities of the taxonomic compositions were calculated using the *vegdist* function, and the compositional differences were visualized by a non-metric multidimensional scaling (nMDS) plot using the *metaMDS* function. Vectors of the environmental variables were fitted onto the nMDS ordination space based on linear relationships, and the coordination significance was evaluated by permutation tests (n = 10 000) using the *envfit* function. To test for correlation between taxonomic composition and sampling season, a Mantel test was performed between the Bray–Curtis dissimilarity matrix of taxonomic composition and a matrix of seasonal distance of the sampling dates using the *mantel* function (Spearman’s rank correlation, permutations = 10 000). Seasonal distance was defined as the number of days between sampling dates, or | 365.25 − (number days) | if the number of days accounted for more than half a year (182 days).

Functional annotation of the extracted prokaryotic sequences was based on Kyoto Encyclopedia of Genes and Genomes (KEGG) Orthology (KO) [36] and was obtained using GhostKOALA [37]. Then, we mapped the predicted KO (K numbers) to KEGG pathways according to the ’Reference hierarchy (KO)’ on the KEGG website (September 2017). We use KEGG pathways in the broad categories of Metabolism, Genetic Information Processing, Environmental Information Processing and Cellular Processes. The abundance of KEGG pathway was obtained by dividing the number of sequences mapped to the pathway by the total number of non-cyanobacterial prokaryotic sequences assigned to any KO. The total abundance was greater than 1.0 because some KOs were assigned to more than one KEGG pathway or category. As with the taxonomic composition, the functional gene compositions were analyzed as follows: the compositional difference was visualized by a nMDS plot based on Bray–Curtis dissimilarity, coordination of the environmental variables to the nMDS ordination was evaluated (permutations = 10 000), and seasonality of the compositional differences was assessed by a Mantel test between the Bray–Curtis dissimilarity and the seasonal distance of the sampling dates (Spearman’s correlation, permutations = 10 000). In addition, correlation between the functional gene and taxonomic compositions was assessed by a Mantel test between the respective Bray–Curtis dissimilarity matrices (Spearman’s correlation, permutations = 10 000).

The KEGG pathways with a relative abundance larger than 0.01 in at least one sample were subjected to hierarchical clustering using the *hclust* function in the *stats* package [33]. The distance between the KEGG pathways was ’1 − (Spearman’s correlation coefficient)’, and the clustering method was ’ward.D2’. The clustering result was visualized in a heatmap generated using the *heatmap*.*2* function in the *gplots* package [38]. A heatmap was also generated with the KEGG modules included in KEGG pathways in the category of amino acid metabolism. We tested Spearman’s correlation between those KEGG pathways and the major taxonomic groups and environmental variables using the *cor*.*test* function in the *stats* package.

We also analyzed KEGG category composition as described above for KEGG pathway composition. Furthermore, we randomly extracted 50 000 prokaryotic sequences assigned to any KO from each sample and determined the Simpson diversity index of KO using the *diversity* function.

## Results

### Sequencing and domain rank composition

Metagenomic sequencing yielded 35 592 379 raw reads, of which 23 780 826 reads with a mean read length of 195 bp passed quality control. Gene prediction resulted in 13 625 130 predicted peptide sequences, of which 6 070 954 were assigned to either a domain (bacteria, archaea, eukaryota) or viruses. The number of sequences per sample is shown in S1 Table.

**Fig 2:**
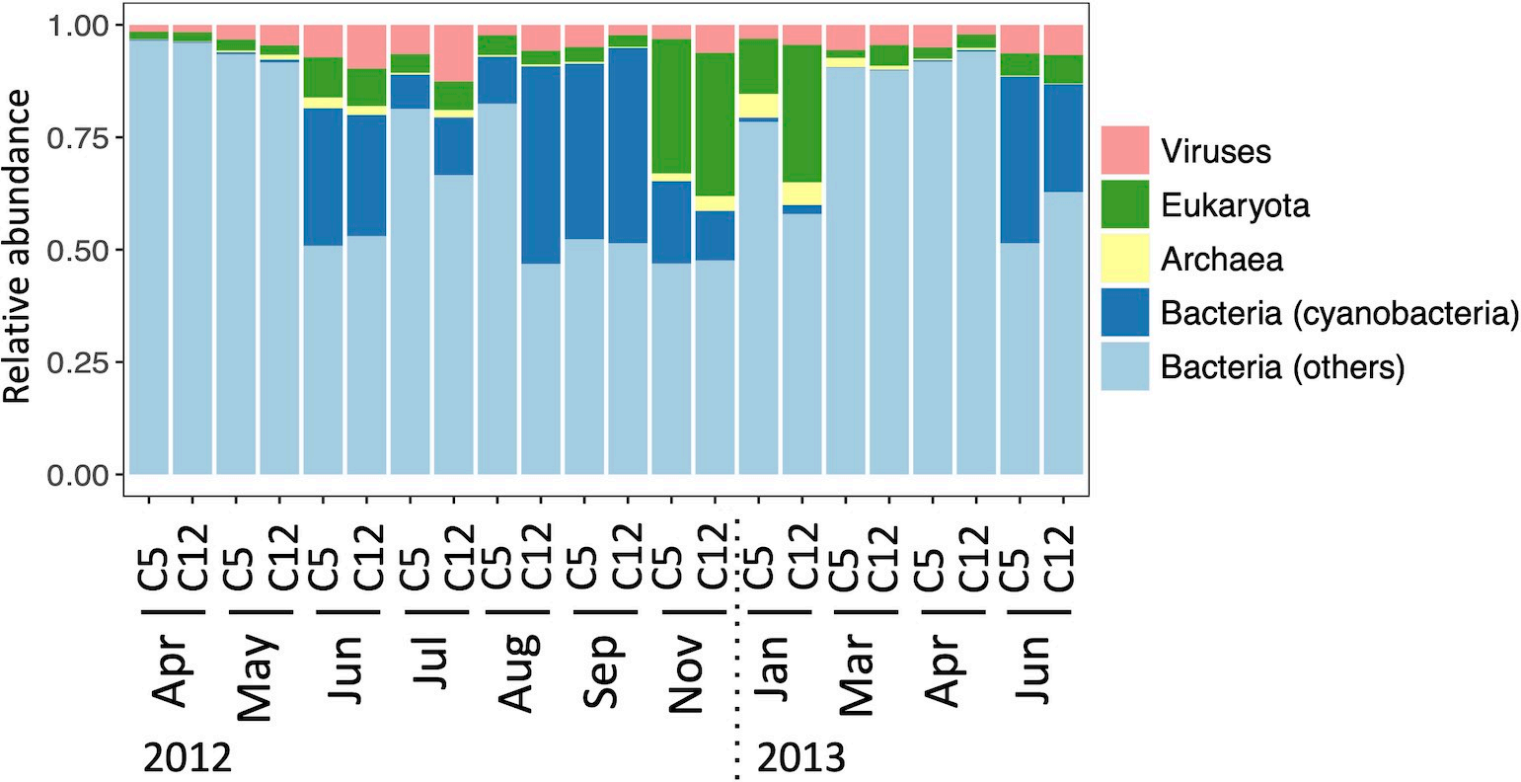
Domain rank composition of predicted peptide sequences. Cyanobacterial sequences are distinguished from other bacterial sequences.

Fig 2 shows the taxonomic composition of the predicted peptide sequences in the domain rank, which includes viruses and distinguishes cyanobacteria and other bacteria. In most samples, the majority (79%–97%) of the taxonomically annotated sequences were attributed to bacteria. In the three exceptions, C5 and C12 in November and C12 in January, a considerable percentage of the sequences was attributed to eukaryota (30%–32%), but bacterial sequences were still more abundant (59%–65%). Among the bacterial sequences, cyanobacterial sequences were particularly abundant from June to November, accounting for 9%–48% of the bacterial sequences during this period. Across all the samples, archaeal and viral sequences were < 0.1%–5% and 1.5%–13%, respectively.

The total number of prokaryotic sequences, excluding cyanobacterial sequences, extracted for further analysis was 4 370 609, and ranged from 97 420 to 397 849 per sample (S1 Table).

### Taxonomic profile of prokaryotic sequences

**Fig 3:**
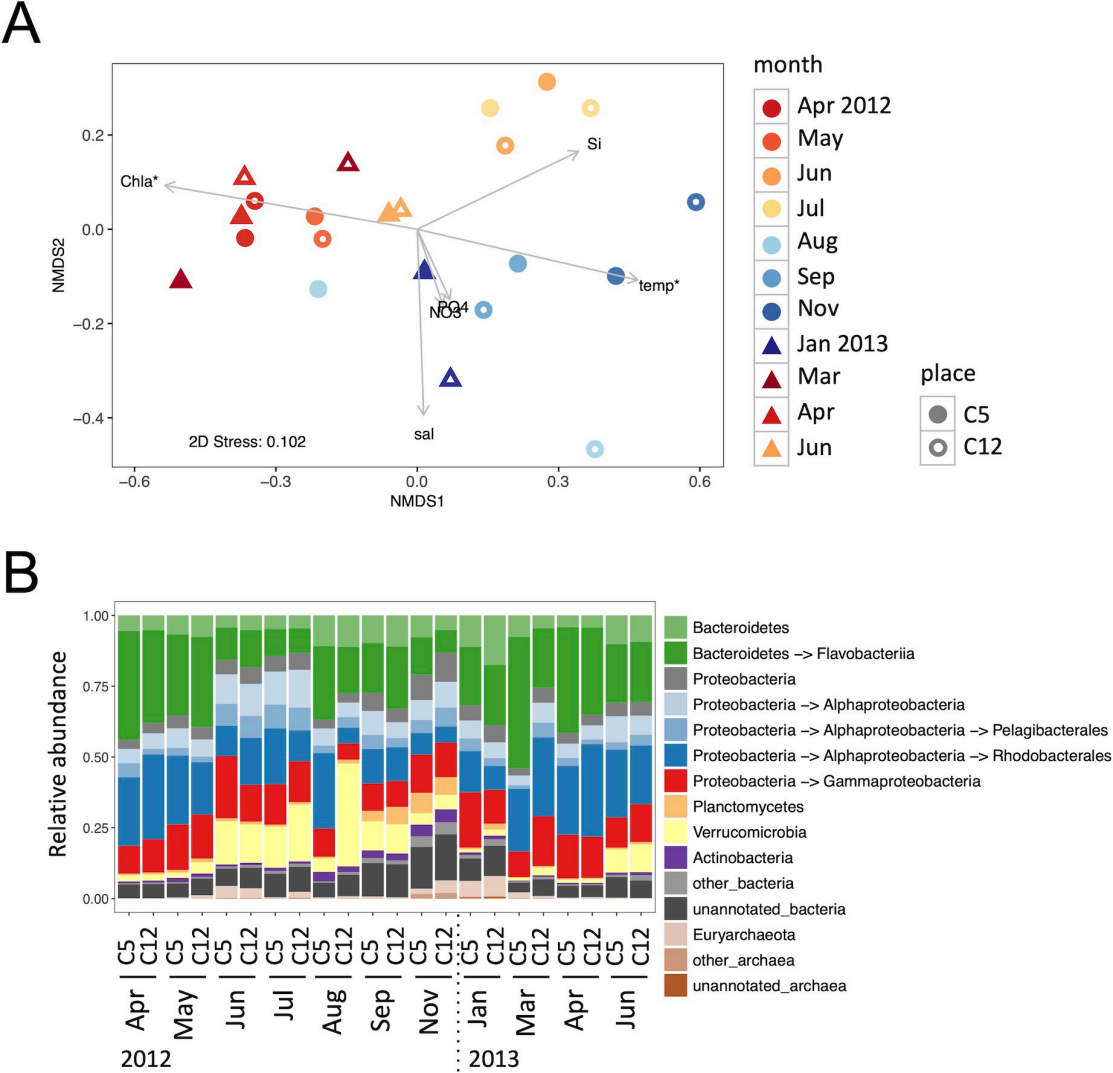
Taxonomic profile of non-cyanobacterial prokaryotic sequences. (A) Nonmetric multidimensional scaling (nMDS) plot of taxonomic composition. Environmental variables are fitted as vectors on the plot; arrow lengths are scaled based on the squared correlation coefficients and the asterisks indicate significant correlations (p < 0.05) with the nMDS ordination. (B) Taxonomic composition using phylum–order rank assignment. Only phyla with max abundance > 0.04 are shown.

Taxonomic composition of the prokaryotic sequences used phylum–order rank assignments, and the compositional variability was visualized by a nMDS plot (Fig 3A). The nMDS plot showed a seasonal trend, with samples from the same or close months tending to have similar compositions regardless of the sampling site and year, although the August samples greatly differed in composition between sampling sites. Seasonality of the taxonomic compositions was confirmed by a Mantel test between Bray–Curtis dissimilarities and seasonal differences in sampling date (r_s_ = 0.46, p < 0.001). *Envfit* analysis found that temperature and Chl *a* concentration were significantly associated with taxonomic composition (p < 0.05).

The phylum–order rank taxonomic composition is shown in Fig 3B. In the spring samples (April and May 2012 and March and April 2013), Flavobacteriia and Rhodobacterales were dominant. In early summer (June and July 2012 and June 2013), Flavobacteriia decreased while Verrucomicrobia and Pelagibacterales increased. The August samples greatly differed between the sampling sites: the C12 sample had abundant Verrucomicrobia, whereas the C5 sample was rich in Flavobacteriia and Rhodobacterales, similar to the spring samples. During the late summer and autumn (September and November 2012), Verrucomicrobia gradually decreased while Planctomycetes and Actinobacteria increased. The number of unannotated bacterial sequences (sequences not assigned to a phylum) also increased during this period. In winter (January 2013), the composition again approached the spring composition, with an increase in Flavobacteriia. Euryarchaeota was relatively abundant in June and November 2012 and January 2013.

### Functional profile of prokaryotic sequences

**Fig 4:**
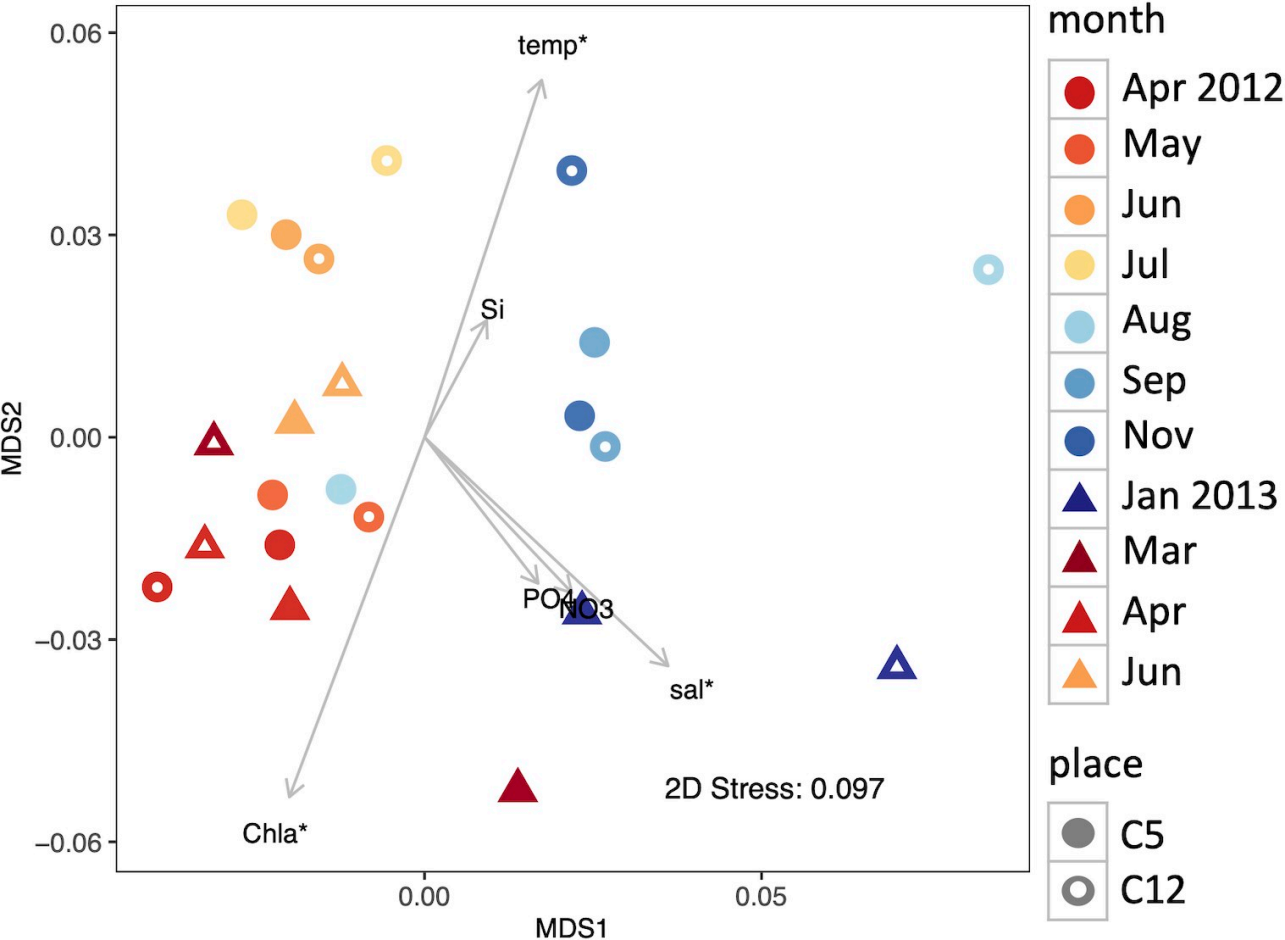
Nonmetric multidimensional scaling (nMDS) plot of KEGG pathway composition of non-cyanobacterial prokaryotic sequences. Environmental variables are fitted as vectors on the plot; arrow lengths are scaled based on the squared correlation coefficients and the asterisks indicate significant correlations (p < 0.05) with the nMDS ordination.

Among the sequences of prokaryotes excluding cyanobacteria, 2 328 421 sequences were assigned based on KO, of which 1 677 853 sequences, which ranged from 37 545 to 160 907 sequences per sample, were further assigned to a KEGG pathway (not all KOs were assigned to a KEGG pathway). The number of those sequences in each sample is shown in S1 Table. Functional gene composition was based on the relative abundance of KEGG pathways, and the compositional variability was visualized by a nMDS plot (Fig 4). Similar to taxonomic composition, the August samples greatly differed between the sampling sites, but the nMDS plot showed a seasonal trend, and seasonality of the compositions was confirmed by a Mantel test (r_s_ = 0.50, p < 0.001). *Envfit* analysis found that temperature, salinity, and Chl *a* concentration were significantly associated with functional gene compositional (p < 0.05). In addition, a Mantel test revealed a significant association between the taxonomic and functional gene compositions (r_s_ = 0.77, p < 0.001).

**Fig 5:**
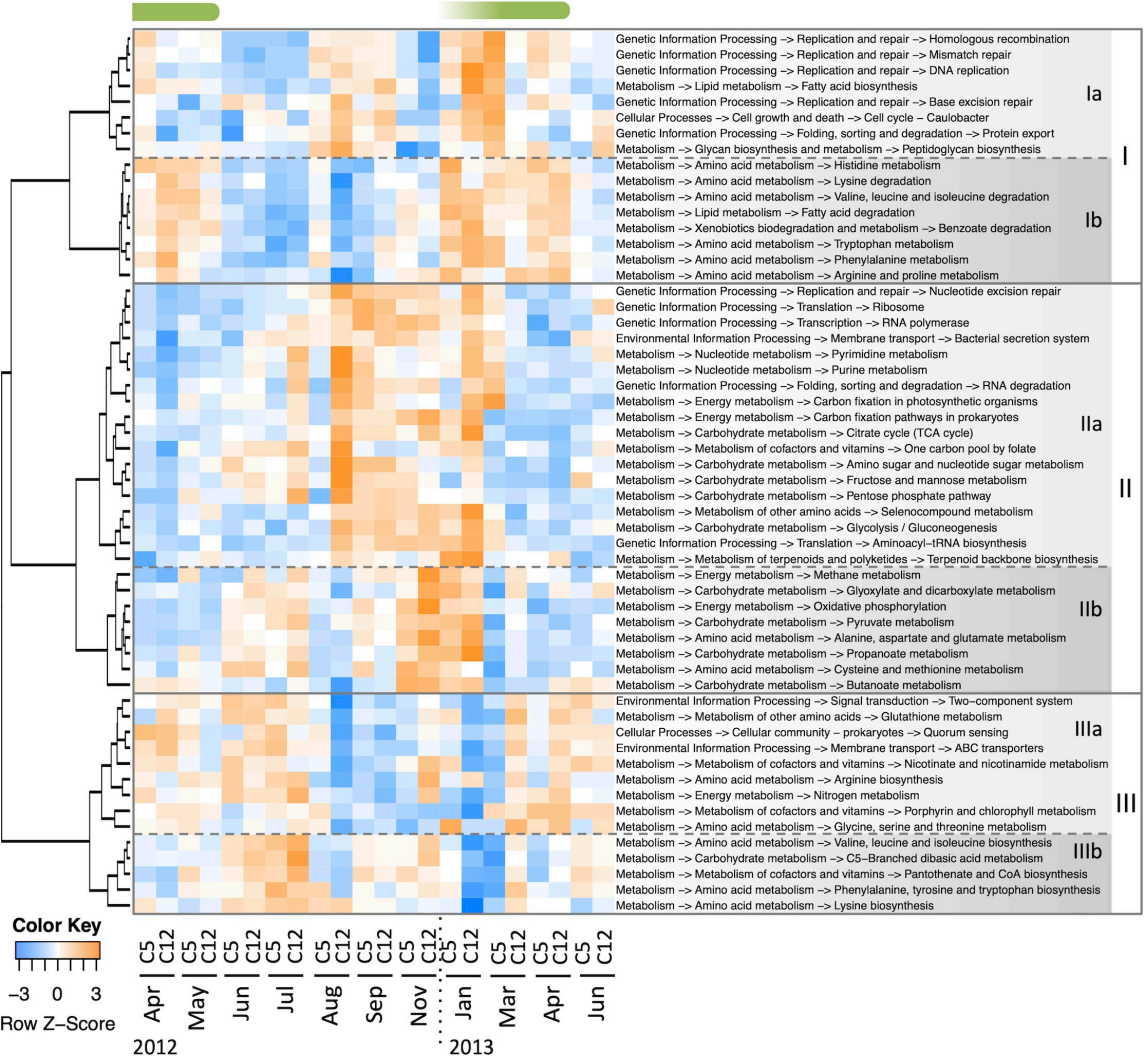
Seasonal patterns of KEGG pathways in non-cyanobacterial prokaryotic sequences. The labels indicate “broad category → KEGG category → KEGG pathway”. Only KEGG pathways with max abundance > 0.01 are shown. The green bars above the heatmap indicate spring phytoplankton blooms.

**Fig 6:**
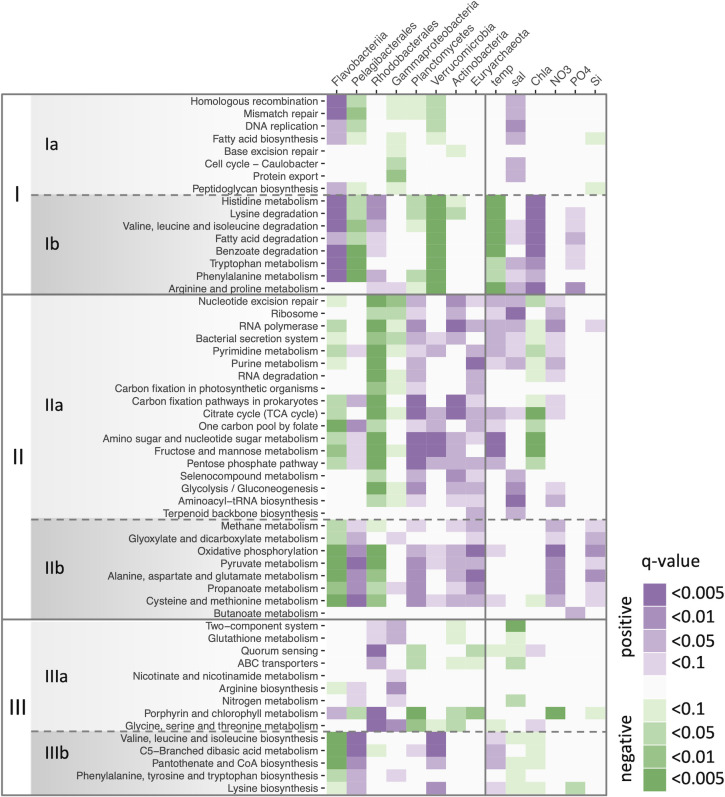
Significance of Spearman’s correlations between KEGG pathways and taxonomic groups or environmental variables. The KEGG pathways are arranged in the same order as in Fig 5.

The functional gene composition is shown in S1 Fig. Based on the increase/decrease pattern in relative abundance, the KEGG pathways clustered into three major groups (Fig 5, I–III). Each group was further divided into two, for a total of six clusters (Fig 5, Ia–IIIb). Fig 6 shows the correlations of these KEGG pathways with the taxonomic groups and environmental variables. The KEGG pathways in cluster I were generally increased in winter and early spring. In particular, those in cluster Ib were positively correlated with Chl *a* concentration and negatively correlated with temperature, and well linked to the spring phytoplankton bloom (Figs 5 and 6). Six out of the eight pathways in cluster Ib were amino acid metabolic pathways. The KEGG pathways in cluster II, conversely, tended to decrease in abundance during the spring bloom; many of the carbohydrate metabolism pathways were included in this cluster. The pathways in cluster IIIa tended to increase in abundant in mid- and post-bloom: from April to July 2012, and March and April 2013 at C12, and June at C5 and C12. The pathways in cluster IIIb increased after the collapse of the bloom in June and July 2012. Cluster IIIb included three amino acid biosynthesis pathways. S2 Fig shows the increase/decrease patterns in abundance of KEGG modules included KEGG pathways of amino acid metabolism. Note that only some parts in the pathways are grouped into modules. These modules were clustered into two groups, those generally increased in early summer (June and July) in 2012 ant those decreased at that period. Many of the modules in the former group, however, were not increased in June 2013.

The KEGG category composition was analyzed in the same way as the KEGG pathways (S3–S6 Figs). The nMDS plot was very similar to that of KEGG pathways, which also indicated seasonality at the KEGG category level.

The Simpson diversity indices of KO were nearly saturated, with only small differences among the samples (0.9986–0.9989), but tended to be slightly lower from summer to winter (<0.9988) than from spring to early summer (>0.9988, S7 Fig). The samples with slightly lower diversity were from September 2012 to March 2013 at C5 and from July 2012 to January 2013 at C12 (S7 Fig), which were generally located on the center to the right side of the nMDS plot (Fig 4). The mean and median ratios of the KO assigned sequences to the total non-cyanobacterial prokaryotic sequences were not significantly different between the 9 samples with slightly lower KO diversity and the other samples (S1 Table, p-values from Welch’s t test and from Mann-Whitney U test were larger than 0.8).

## Discussion

### Seasonal changes in community structure were accompanied by changes in overall functional profile

The functional gene composition of the non-cyanobacterial prokaryotic community seasonally varied with taxonomic composition (Figs 3A and 4; Mantel tests). The functional composition was associated with Chl *a* concentration, temperature, and salinity. These environmental factors have often been reported to be associated with seasonal variation in microbial community structure [6,8,15], and were also associated with seasonal variation in 16S community composition in Sendai Bay [28]. The taxonomic composition of the metagenomes in this study did not show a significant association with salinity, probably because of the low resolution of taxonomic assignment (phylum/order). As our data were only available for a little over a year, we need more years of observation to see if the results are annual repeating patterns. However, the taxonomic composition (Fig 3B) was consistent with the typical seasonal pattern in a temperate ocean [10], and the phytoplankton community composition and environmental variables during the sampling period followed the usual seasonal pattern in this bay [26,27]. Therefore, the seasonal changes in functional gene composition observed in this study are also expected to represent some general trends in temperate coastal regions.

In the previous studies that reported seasonal differences in metagenomic functional profiles, photosynthesis-related genes of cyanobacteria and picophytoplankton contributed remarkably to the differences [15,24]. Our metagenomic data also showed large increases and decreases in the sequences of those organisms (Fig 2). We excluded their sequences to focus on the heterotrophs and still found seasonal variation in the functional profiles. This highlights competition effects among functionally different taxa in shaping the seasonal cycle of the community structure of non-cyanobactterial prokaryotes.

### Seasonal patterns in functional profiles

During the spring phytoplankton bloom, predicted genes potentially related to several amino acid metabolism pathways increased in abundance (cluster Ib in Fig 5), and their seasonal patterns were linked to Chl *a* concentration (Fig 6). Free and combined amino acids (e.g., oligopeptides and proteins) are essential sources of carbon, nitrogen, and energy for marine heterotrophic microbes [39]. Although various organisms release and utilize amino acids, the main source of release is phytoplankton and the main utilizer is bacteria [39,40]. Actively growing phytoplankton promote amino acid production [39,41], and bacteria quickly utilize those that are biologically available [40,42,43]. Therefore, phytoplankton blooms may have increased the supply of various amino acids, which led to an increase of prokaryotic taxa capable of extensively utilizing the amino acids.

Although many of the amino acid metabolic pathways increased during the phytoplankton bloom, several amino acid pathways showed different patterns (Fig 5). These included the pathways of the dominant amino acids in seawater: Gly, Asp, Glu, Ala, and Ser [44]. That is, the KEGG pathways that increased during the phytoplankton bloom were those of amino acids that are normally found at relatively low concentrations. The difference in pattern may be related to changes in the composition of amino acids available in the environment. Released amino acids vary in composition and proportions among phytoplankton species, which leads to different community structures of heterotrophic prokaryotes [45]. Supplies from other sources, such as bacterial cell lysis, would also affect the available amino acid composition, especially during periods of low phytoplankton population [46]. Some amino acids metabolic pathways specifically classified as its biosynthesis were increased in early summer in 2012 (cluster IIIb in Fig 5), and KEGG modules of biosynthesis generally increased at that period, too (S2 Fig). Since there seems to be many unidentified steps in bacterial amino acids biosynthesis pathways [47], the KOs considered here may be only a small part of the biosynthesis-related genes. This KEGG-based results, however, provide the possibility that the importance of some amino acids biosynthesis was relatively high in the early summer.

In contrast to many amino acid metabolic pathways, carbon metabolism pathways generally increased in abundances of related genes in the non-bloom phase (cluster II in Fig 5). At the KEGG category level, similarly, “carbon metabolism” increased in abundance in the non-bloom phase (S5 Fig). The fundamental pathways related to respiration indicate corbon usage for energy souce. When considered together with Ribosome and RNA polymerase in cluster IIa, it seems that more basic functional genes became high in the ratio during that period. The other carbon metabolism pathways suggest that the relative importance of carbohydrate in biosynthesis was also increased. This result seems to be consistent with the observation in the Southern Ocean that glucose incorporation and significance of dissolved carbohydrates as substrates for heterotrophic picoplankton growth were enhanced during the low Chl *a* period, when protein supply was reduced [48]. During seasons of low phytoplankton production, there were probably fewer available amino acids, which may have increased the reliance of the prokaryotic community on carbon metabolism.

The KEGG pathways in cluster IIIa of Fig 5 increased in abundances of related genes during and after the phytoplankton bloom, and those patterns lagged behind Chl *a* concentration. During the growing phase of a bloom, living phytoplankton release soluble, labile, low molecular weight organic matters by exudate; alternatively, during the waning phase, dying phytoplankton release macromolecules and particulate materials primarily as a result of cell lysis [13]. Considering the timing of the increase/decrease in abundance, the pathways in cluster Ib may be associated with the former phase and the pathways in cluster IIIa with the latter. Consistent with this, cluster IIIa includes KEGG pathways that may be associated with a copiotrophic, particle-attached lifestyle. Biogenic particles and aggregates are generally rich in organic matter, and colonizing those surfaces is a typical lifestyle for copiotrophic taxa [49]. Sensing and responses to environmental changes, including inter/intra-species communication, are important for surface-associated lifestyles, and they are facilitated by two-component signal transduction systems and quorum sensing [50]. The overall abundances of genes mapped to the KEGG categories “signal transduction” and “cellular community” were also relatively high during and after the phytoplankton bloom (S5 Fig). Metagenomic studies have found that particle-associated communities are usually enriched in genes for functions such as signaling and cell–cell interactions [50]. Given the fraction size of the present study (0.8–5.0 μm), the main particulate materials may be fine organic detritus and aggregates. For example, transparent exopolymer particles (TEPs) that form from phytoplankton-derived precursors and become abundant during phytoplankton blooms can be rapidly colonized and utilized by bacteria and may alter the bacterial community structure [51,52].

In addition to composition, the diversity of functional genes has been used to study microbial communities [17,53]. The diversity indices of KO were very large, with only small differences among the samples, but the diversity tended to be particularly high during and after the phytoplankton bloom when the abundances of genes for KEGG pathways in cluster IIIa were increased (Figs 5 and S7). The differences in KO diversity did not show an association with the proportion of sequences mapped to KOs (see Results section), suggesting that it likely reflects a difference in the overall functional diversity rather than annotation bias. The high KO diversity indicated diverse species and metabolic capabilities to use phytoplankton-derived nutrients that are a mixture of various compounds. The relatively high abundance of ABC transporters in this period also indicates uptake of a diverse range of substrates (Fig 5). Seemingly, this result is not consistent with that of a previous study, which found a negative correlation between functional gene diversity and primary production in surface seawater [17]. The most likely reason for this discrepancy is that, in this study, we excluded cyanobacterial sequences that would contribute to genetic diversity during a low Chl *a* period. Another probable explanation is the difference in fraction sizes. We examined a larger fraction that included prokaryotes colonizing small particles, and they may have contributed to the genetic diversity during the high Chl *a* period.

### Differences between sampling sites

Differences in the taxonomic and functional gene compositions between the sampling sites at the same time point were generally small compared to the seasonal differences, but there were substantial differences in August 2012 (Figs 3A and 4). The cyanobacterial sequence abundance also greatly differed at that time, and was much less at C5 than C12 (Fig 2). This is consistent with the previous report, which found that there was a short-term drop in cyanobacterial cell abundance in C5 [26]. This large difference in composition indicates that there were large environmental differences within Sendai Bay at that time. In particular, the functional composition at C5, which was similar to those in the high Chl *a* period (Fig 4), indicates that C5 was a more nutrient-rich environment than C12. One possible cause of this is river water discharge. Low salinity and high silicate concentrations were observed in surface waters around early summer, especially at the coastal C5 site, and were thought to be due to river water discharge (Table 1) [26,27]. The presumed silicate supply seems to have resulted in sporadic increases of some diatom species from summer to fall [27]. Thus, in August at C5, river water discharge may have led to a nutrient-rich environment and an increase of copiotrophic prokaryotes.

Regarding the locational differences in Sendai Bay, Kakehi et al. reported that the mechanism of phytoplankton bloom initiation would vary with bottom depth [54]. They observed that the increase in Chl *a* concentration occurred earlier in the shallow area than in the deeper area and occurred during the winter convective season. This was thought to have occurred because in the shallow areas, vertical mixing reached the bottom and phytoplankton cells remained in the region of high light intensity, even before stratification was established [54]. In 2013, the increase of Chl *a* concentration was approximately a month earlier at the shallower C5 site than at C12, and the bloom onset was thought to be in late January at C5 and mid-March at C12 (Table 1) [26,27]. In January and March 2013, there were relatively large differences between the functional compositions of C5 and C12, and the composition at C12 greatly changed from January to March (Fig 4). This may have reflected the difference in the process of spring bloom initiation.

## Conclusions

This study provided a functional metagenomics perspective on seasonal change in coastal non-cyanobacterial prokaryotic communities. The functional profiles seasonally varied, and the variation seemed to reflect changes in available nutrient concentration and composition associated with the seasonal succession of the phytoplankton population. Our results support that the seasonal cycle of prokaryotic community structure is driven mainly by changes in trophic condition and accompanied with changes in overall community function. Metagenomics analyses provide prediction for the types of functional genes that are important in the environment based on the gene content; however, these analyses need to be complemented by metatranscriptomic, metaproteomic, and other experimental approaches that provide information on microbial activity to better elucidate community dynamics. Nevertheless, our findings from seasonal, time-series metagenomes provide fundamental information for future studies on marine microbial community dynamics.

## Supporting information

S1 Fig Relative abundances of KEGG pathways in non-cyanobacterial prokaryotic sequences. The labels indicate “broad category → KEGG category → KEGG pathway”. Only KEGG pathways with max abundance > 0.02 are shown. As some genes are assigned to more than one pathway, the total is greater than 1.0.
(PDF)

S2 Fig Seasonal patterns of KEGG modules included in amino acids metabolism pathways in non-cyanobacterial prokaryotic sequences. Only KEGG modules with max abundance > 0.001 are shown. The green bars above the heatmap indicate spring phytoplankton blooms.
(PDF)

S3 Fig Nonmetric multidimensional scaling (nMDS) plot of KEGG category composition of non-cyanobacterial prokaryotic sequences. Environmental variables are fitted as vectors on the plot; arrow lengths are scaled based on the squared correlation coefficients, and the asterisks indicate significant correlations (p < 0.05) with the nMDS ordination.
(PDF)

S4 Fig Relative abundances of KEGG categories in non-cyanobacterial prokaryotic sequences. The labels indicate “broad category → KEGG category”. Only KEGG categories with max abundance > 0.02 are shown. As some genes are assigned to more than one category, the total is greater than 1.0.
(PDF)

S5 Fig Seasonal patterns of KEGG categories in non-cyanobacterial prokaryotic sequences. The labels indicate “broad category → KEGG category”. Only KEGG categories with max abundance > 0.01 are shown. The green bars above the heatmap indicate spring phytoplankton blooms.
(PDF)

S6 Fig Significance of Spearman’s correlations between KEGG categories and taxonomic groups or environmental variables. The KEGG categories are arranged in the same order as in S4 Fig.
(PDF)

S7 Fig Simpson diversity index of the KO using 50 000 randomly extracted sequences per sample. The plot color and shape are the same as in Fig 4.
(PDF)

S1 Table Number of sequences in each sample. The values in brackets indicate the percentage relative to the total number of sequences of prokaryotes other than cyanobacteria.
(PDF)

## References

[1] Azam F, Fenchel T, Field JG, Gray JS, Meyer-Reil LA, Thingstad F. The ecological role of water-column microbes in the sea. Mar Ecol Prog Ser. 1983;10: 257–263. doi: 10.3354/meps010257

[2] Cole JJ, Findlay S, Pace ML. Bacterial production in fresh and saltwater ecosystems: a cross-system overview. Mar Ecol Prog Ser. 1988;43: 1–10. doi: 10.3354/meps043001

[3] Azam F, Malfatti F. Microbial structuring of marine ecosystems. Nat Rev Microbiol. 2007;5: 782–791. doi: 10.1038/nrmicro1747 17853906

[4] Zinger L, Amaral-Zettler LA, Fuhrman JA, Horner-Devine MC, Huse SM, Welch DBM, et al. Global patterns of bacterial beta-diversity in seafloor and seawater ecosystems. PLoS One. 2011;6: e24570. doi: 10.1371/journal.pone.0024570 21931760 PMC3169623

[5] Chow CET, Sachdeva R, Cram JA, Steele JA, Needham DM, Patel A, et al. Temporal variability and coherence of euphotic zone bacterial communities over a decade in the Southern California Bight. ISME J. 2013;7: 2259–2273. doi: 10.1038/ismej.2013.122 23864126 PMC3834854

[6] Gilbert JA, Steele JA, Caporaso JG, Steinbrück L, Reeder J, Temperton B, et al. Defining seasonal marine microbial community dynamics. ISME J. 2012;6: 298–308. doi: 10.1038/ismej.2011.107 21850055 PMC3260500

[7] Giovannoni SJ, Vergin KL. Seasonality in ocean microbial communities. Science 2012;335: 671–676. doi: 10.1126/science.1198078 22323811

[8] Cram JA, Chow CET, Sachdeva R, Needham DM, Parada AE, Steele JA, et al. Seasonal and interannual variability of the marine bacterioplankton community throughout the water column over ten years. ISME J. 2015;9: 563–580. doi: 10.1038/ismej.2014.153 25203836 PMC4331575

[9] Lindh MV, Sjöstedt J, Andersson AF, Baltar F, Hugerth LW, Lundin D, et al. Disentangling seasonal bacterioplankton population dynamics by high‐frequency sampling. Environ Microbiol. 2015;17: 2459–2476. doi: 10.1111/1462-2920.12720 25403576

[10] Bunse C, Pinhassi J. Marine bacterioplankton seasonal succession dynamics. Trends Microbiol. 2017;25: 494–505. doi: 10.1016/j.tim.2016.12.013 28108182

[11] Teeling H, Fuchs BM, Becher D, Klockow C, Gardebrecht A, Bennke CM, et al. Substrate-controlled succession of marine bacterioplankton populations induced by a phytoplankton bloom. Science 2012;336: 608–611. doi: 10.1126/science.1218344 22556258

[12] Teeling H, Fuchs BM, Bennke CM, Krueger K, Chafee M, Kappelmann L, et al. Recurring patterns in bacterioplankton dynamics during coastal spring algae blooms. Elife 2016;5: e11888. doi: 10.7554/eLife.11888 27054497 PMC4829426

[13] Buchan A, LeCleir GR, Gulvik CA, González JM. Master recyclers: features and functions of bacteria associated with phytoplankton blooms. Nat Rev Microbiol. 2014;12: 686–698. doi: 10.1038/nrmicro3326 25134618

[14] Hunt DE, David LA, Gevers D, Preheim SP, Alm EJ, Polz MF. Resource partitioning and sympatric differentiation among closely related bacterioplankton. Science 2008;320: 1081–1085. doi: 10.1126/science.1157890 18497299

[15] Ward CS, Yung CM, Davis KM, Blinebry SK, Williams TC, Johnson ZI, et al. Annual community patterns are driven by seasonal switching between closely related marine bacteria. ISME J. 2017;11: 1412–1422. doi: 10.1038/ismej.2017.4 28234350 PMC5437356

[16] Sharpton TJ. An introduction to the analysis of shotgun metagenomic data. Front Plant Sci. 2014;5: 209. doi: 10.3389/fpls.2014.00209 24982662 PMC4059276

[17] Raes J, Letunic I, Yamada T, Jensen LJ, Bork P. Toward molecular trait‐based ecology through integration of biogeochemical, geographical and metagenomic data. Mol Syst Biol. 2011;7: 473. doi: 10.1038/msb.2011.6 21407210 PMC3094067

[18] DeLong EF, Preston CM, Mincer T, Rich V, Hallam SJ, Frigaard NU, et al. Community genomics among stratified microbial assemblages in the ocean’s interior. Science. 2006;311: 496–503. doi: 10.1126/science.1120250 16439655

[19] Smith MW, Zeigler Allen L, Allen AE, Herfort L, Simon HM. Contrasting genomic properties of free-living and particle-attached microbial assemblages within a coastal ecosystem. Front Microbiol. 2013;4: 120. doi: 10.3389/fmicb.2013.00120 23750156 PMC3668451

[20] Ganesh S, Parris DJ, DeLong EF, Stewart FJ. Metagenomic analysis of size-fractionated picoplankton in a marine oxygen minimum zone. ISME J. 2014;8: 187–211. doi: 10.1038/ismej.2013.144 24030599 PMC3869020

[21] Burke C, Steinberg P, Rusch D, Kjelleberg S, Thomas T. Bacterial community assembly based on functional genes rather than species. Proc Natl Acad Sci U S A. 2011;108: 14288–14293. doi: 10.1073/pnas.1101591108 21825123 PMC3161577

[22] Haggerty JM, Dinsdale EA. Distinct biogeographical patterns of marine bacterial taxonomy and functional genes. Glob Ecol Biogeogr. 2017;26: 177–190. doi: 10.1111/geb.12528

[23] Galand PE, Pereira O, Hochart C, Auguet JC, Debroas D. A strong link between marine microbial community composition and function challenges the idea of functional redundancy. ISME J. 2018;12: 2470–2478. doi: 10.1038/s41396-018-0158-1 29925880 PMC6155072

[24] Gilbert JA, Field D, Swift P, Thomas S, Cummings D, Temperton B, et al. The taxonomic and functional diversity of microbes at a temperate coastal site: a ’multi-omic’ study of seasonal and diel temporal variation. PLoS One. 2010;5: e15545. doi: 10.1371/journal.pone.0015545 21124740 PMC2993967

[25] Kataoka T, Yamaguchi H, Sato M, Watanabe T, Taniuchi Y, Kuwata A, et al. Seasonal and geographical distribution of near-surface small photosynthetic eukaryotes in the western North Pacific determined by pyrosequencing of 18S rDNA. FEMS Microbiol Ecol. 2017;93: fiw229. doi: 10.1093/femsec/fiw229 27810875

[26] Taniuchi Y, Watanabe T, Kakehi S, Sakami T, Kuwata A. Seasonal dynamics of the phytoplankton community in Sendai Bay, northern Japan. J Oceanogr. 2017;73: 1–9. doi: 10.1007/s10872-015-0334-0

[27] Watanabe T, Taniuchi Y, Kakehi S, Sakami T, Kuwata A. Seasonal succession in the diatom community of Sendai Bay, northern Japan, following the 2011 off the Pacific coast of Tohoku earthquake. J Oceanogr. 2017;73: 133–144. doi: 10.1007/s10872-016-0387-8

[28] Sakami T, Watanabe T, Kakehi S. Seasonal Dynamics of Bacterial Community Composition in Coastal Seawater at Sendai Bay, Japan. In: Gojobori T., Wada T., Kobayashi T., Mineta K, editors. Marine Metagenomics. Singapore: Springer; 2019. pp. 137–147. doi: 10.1007/978-981-13-8134-8_9

[29] Gordon A, Hannon GJ. Fastx-toolkit. FASTQ/A short-reads preprocessing tools; 2010. Available from: http://hannonlab.cshl.edu/fastx_toolkit.

[30] Niu B, Fu L, Sun S, Li W. Artificial and natural duplicates in pyrosequencing reads of metagenomic data. BMC bioinformatics. 2010;11: 187. doi: 10.1186/1471-2105-11-187 20388221 PMC2874554

[31] Zhu W, Lomsadze A, Borodovsky M. Ab initio gene identification in metagenomic sequences. Nucleic Acids Res. 2010;38: e132. doi: 10.1093/nar/gkq275 20403810 PMC2896542

[32] Huson DH, Beier S, Flade I, Górska A, El-Hadidi M, Mitra S, et al. MEGAN community edition-interactive exploration and analysis of large-scale microbiome sequencing data. PLoS Comput Biol. 2016;1: e1004957. doi: 10.1371/journal.pcbi.1004957 27327495 PMC4915700

[33] R Core Team. R: A language and environment for statistical computing; 2019. Available from: https://www.R-project.org/.

[34] Oksanen J, Blanchet FG, Friendly M, Kindt R, Legendre P, McGlinn D, et al. vegan: Community Ecology Package. R package version 2.5–6; 2019. Available from: https://CRAN.R-project.org/package=vegan.

[35] Giovannoni SJ. SAR11 bacteria: the most abundant plankton in the oceans. Ann Rev Mar Sci. 2017;9: 231–255. doi: 10.1146/annurev-marine-010814-015934 27687974

[36] Kanehisa M, Goto S. KEGG: kyoto encyclopedia of genes and genomes. Nucleic Acids Res. 2000;28: 27–30. doi: 10.1093/nar/28.1.27 10592173 PMC102409

[37] Kanehisa M, Sato Y, Morishima K. BlastKOALA and GhostKOALA: KEGG tools for functional characterization of genome and metagenome sequences. J Mol Biol. 2016;428: 726–731. doi: 10.1016/j.jmb.2015.11.006 26585406

[38] Warnes GR, Bolker B, Bonebakker L, Gentleman R, Huber W, Liaw A, et al. gplots: Various R programming tools for plotting data. R package version 3.0.3; 2020. Available from: https://CRAN.R-project.org/package=gplots.

[39] Berman T, Bronk DA. Dissolved organic nitrogen: a dynamic participant in aquatic ecosystems. Aquat Microb Ecol. 2003;31: 279–305. doi: 10.3354/ame031279

[40] Fuhrman J. Close coupling between release and uptake of dissolved free amino acids in seawater studied by an isotope dilution approach. Mar Ecol Prog Ser. 1987;37: 45–52. doi: 10.3354/meps037045

[41] Hammer KD, Kattner G. Dissolved free amino acids in the marine environment: a carbon to nitrogen ratio shift during diatom blooms. Mar Ecol Prog Ser. 1986;31: 35–45. doi: 10.3354/meps031035

[42] Casey JR, Falkowski PG, Karl DM. Substrate selection for heterotrophic bacterial growth in the sea. Mar Chem. 2015;177: 349–356. doi: 10.1016/j.marchem.2015.06.032

[43] Uchimiya M, Motegi C, Nishino S, Kawaguchi Y, Inoue J, Ogawa H, et al. Coupled response of bacterial production to a wind-induced fall phytoplankton bloom and sediment resuspension in the Chukchi Sea Shelf, Western Arctic Ocean. Front Mar Sci. 2016;3: 231.

[44] Hubberten U, Lara RJ, Kattner G. Amino acid composition of seawater and dissolved humic substances in the Greenland Sea. Mar Chem. 1994;45: 121–128. doi: 10.1016/0304-4203(94)90096-5

[45] Sarmento H, Romera-Castillo C, Lindh M, Pinhassi J, Sala MM, Gasol JM, et al. Phytoplankton species‐specific release of dissolved free amino acids and their selective consumption by bacteria. Limnol Oceanogr. 2013;58: 1123–1135. doi: 10.4319/lo.2013.58.3.1123

[46] Middelboe M, Jørgensen NO. Viral lysis of bacteria: an important source of dissolved amino acids and cell wall compounds. J Mar Biol Assoc UK. 2006;86: 605–612. doi: 10.1017/S0025315406013518

[47] Price MN, Zane GM, Kuehl JV, Melnyk RA, Wall JD, Deutschbauer AM, et al. Filling gaps in bacterial amino acid biosynthesis pathways with high-throughput genetics. PLoS genetics. 2018;14: e1007147. doi: 10.1371/journal.pgen.1007147 29324779 PMC5764234

[48] Simon M, Rosenstock B. Different coupling of dissolved amino acid, protein, and carbohydrate turnover to heterotrophic picoplankton production in the Southern Ocean in austral summer and fall. Limnol Oceanogr. 2007;52: 85–95. doi: 10.4319/lo.2007.52.1.0085

[49] Lauro FM, McDougald D, Thomas T, Williams TJ, Egan S, Rice S, et al. The genomic basis of trophic strategy in marine bacteria. Proc Natl Acad Sci U S A. 2009;106: 15527–15533. doi: 10.1073/pnas.0903507106 19805210 PMC2739866

[50] Dang H, Lovell CR. Microbial surface colonization and biofilm development in marine environments. Microbiol Mol Biol Rev. 2016;80: 91–138. doi: 10.1128/MMBR.00037-15 26700108 PMC4711185

[51] Passow U. Transparent exopolymer particles (TEP) in aquatic environments. Prog Oceanogr. 2002;55: 287–333. doi: 10.1016/S0079-6611(02)00138-6

[52] Taylor JD, Cottingham SD, Billinge J, Cunliffe M. Seasonal microbial community dynamics correlate with phytoplankton-derived polysaccharides in surface coastal waters. ISME J. 2014;8: 245–248. doi: 10.1038/ismej.2013.178 24132076 PMC3869024

[53] Yang C, Li Y, Zhou Y, Lei X, Zheng W, Tian Y, et al. A comprehensive insight into functional profiles of free-living microbial community responses to a toxic Akashiwo sanguinea bloom. Sci Rep. 2016;6: 34645. doi: 10.1038/srep34645 27703234 PMC5050414

[54] Kakehi S, Ito SI, Kuwata A, Saito H, Tadokoro K. Phytoplankton distribution during the winter convective season in Sendai Bay, Japan. Cont Shelf Res. 2015;97: 43–53. doi: 10.1016/j.csr.2015.02.005

