# Supplementary figures and images for: Metagenomic analysis provides functional insights into seasonal change of a non-cyanobacterial prokaryotic community in temperate coastal waters
Source: PLoS One. 2021 Oct 12;16(10):e0257862. doi: 10.1371/journal.pone.0257862 (PMC8509957; doi:10.1371/journal.pone.0257862)

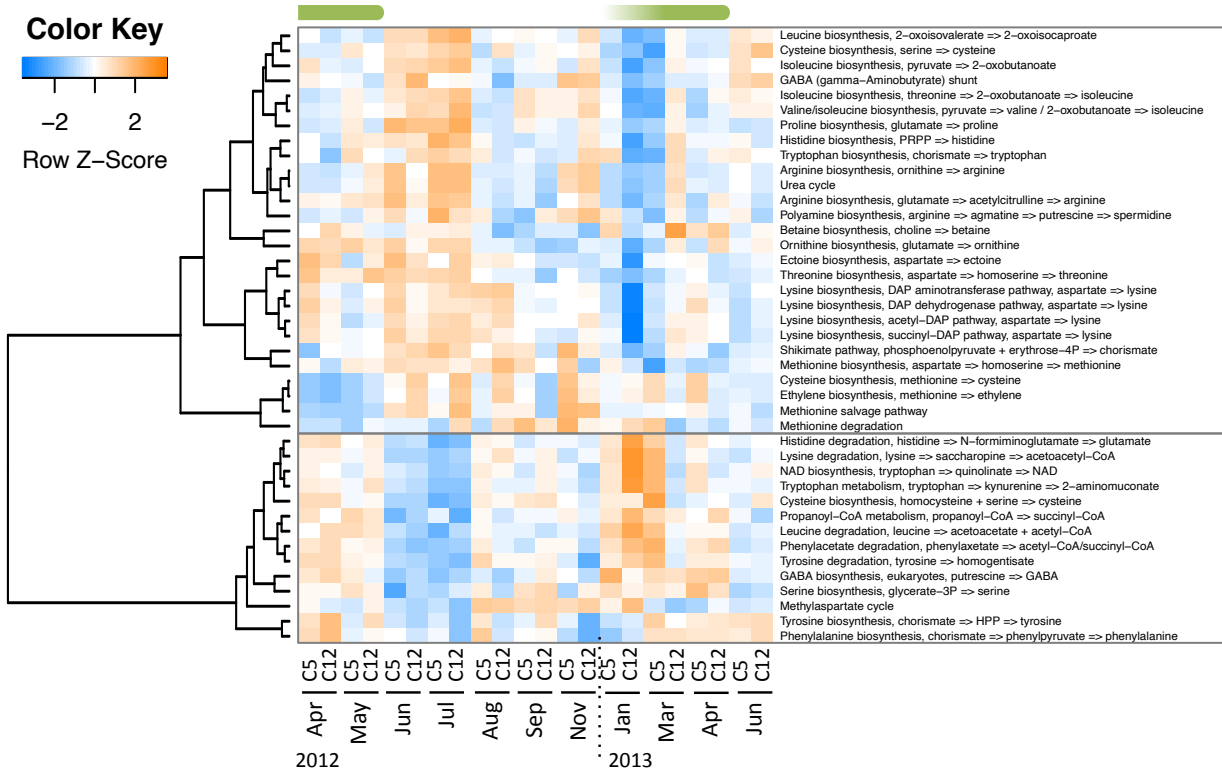

Supplement: S2 Fig — Only KEGG modules with max abundance > 0.001 are shown. The green bars above the heatmap indicate spring phytoplankton blooms. (PDF) [file pone.0257862.s002.pdf]

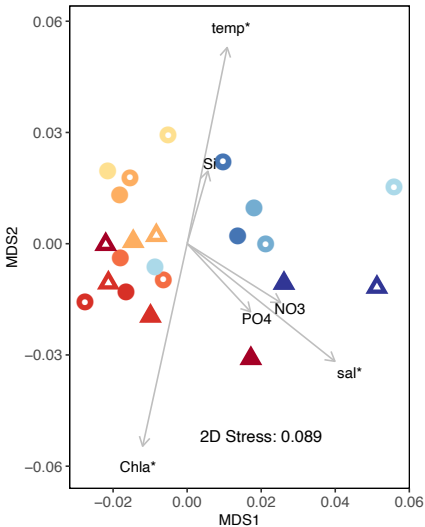

month

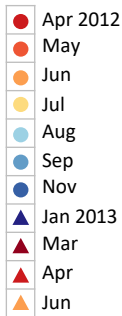

Supplement: S3 Fig — Environmental variables are fitted as vectors on the plot; arrow lengths are scaled based on the squared correlation coefficients, and the asterisks indicate significant correlations (p < 0.05) with the nMDS ordination. (PDF) [file pone.0257862.s003.pdf]

Color Key

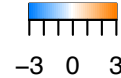

Row Z-Score

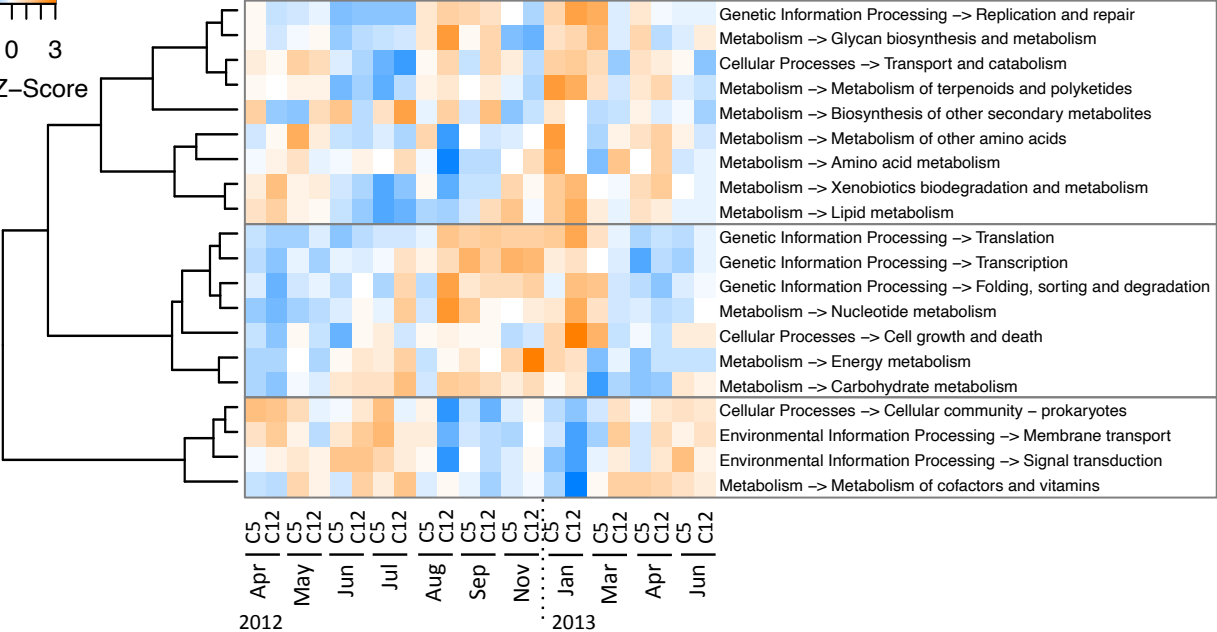

Supplement: S5 Fig — The labels indicate “broad category → KEGG category”. Only KEGG categories with max abundance > 0.01 are shown. The green bars above the heatmap indicate spring phytoplankton blooms. (PDF) [file pone.0257862.s005.pdf]

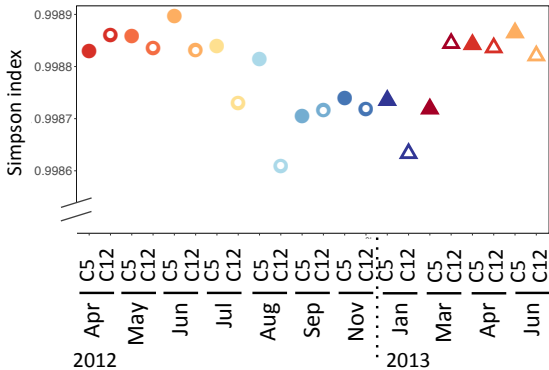

Supplement: S7 Fig — The plot color and shape are the same as in Fig 4. (PDF) [file pone.0257862.s007.pdf]
