# Supplementary material for: Metagenomic analysis provides functional insights into seasonal change of a non-cyanobacterial prokaryotic community in temperate coastal waters
Source: PLoS One. 2021 Oct 12;16(10):e0257862. doi: 10.1371/journal.pone.0257862 (PMC8509957; doi:10.1371/journal.pone.0257862)
Supplement: S4 Fig — The labels indicate “broad category → KEGG category”. Only KEGG categories with max abundance > 0.02 are shown. As some genes are assigned to more than one category, the total is greater than 1.0. (PDF) [file pone.0257862.s004.pdf]

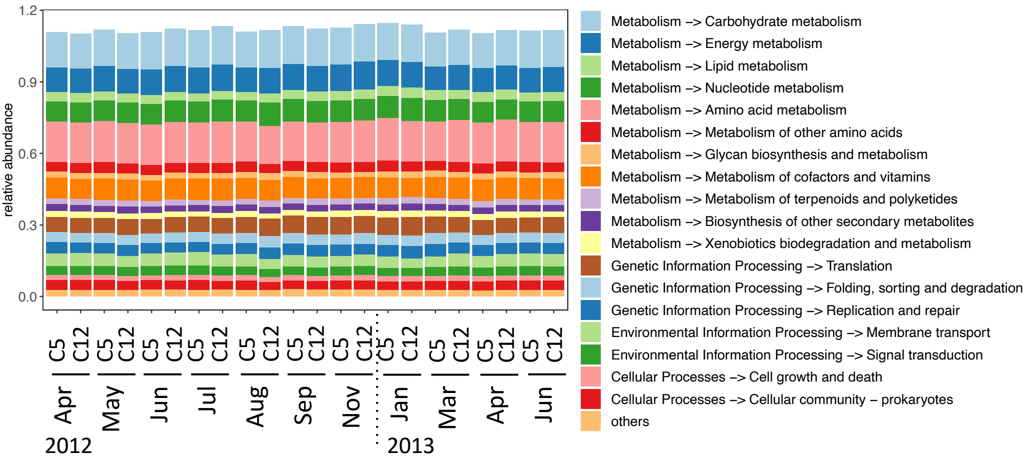

relative abundance
0.0
0.3
0.6
0.9
1.2
C5 C12 C5 C12 C5 C12 C5 C12 C5 C12 C5 C12 C5 C12 C5 C12 C5 C12 C5 C12 C5 C12
Apr May Jun Jul Aug Sep Nov Jan Mar Apr Jun
2012
2013
Metabolism –> Carbohydrate metabolism
Metabolism –> Energy metabolism
Metabolism –> Lipid metabolism
Metabolism –> Nucleotide metabolism
Metabolism –> Amino acid metabolism
Metabolism –> Metabolism of other amino acids
Metabolism –> Glycan biosynthesis and metabolism
Metabolism –> Metabolism of cofactors and vitamins
Metabolism –> Metabolism of terpenoids and polyketides
Metabolism –> Biosynthesis of other secondary metabolites
Metabolism –> Xenobiotics biodegradation and metabolism
Genetic Information Processing –> Translation
Genetic Information Processing –> Folding, sorting and degradation
Genetic Information Processing –> Replication and repair
Environmental Information Processing –> Membrane transport
Environmental Information Processing –> Signal transduction
Cellular Processes –> Cell growth and death
Cellular Processes –> Cellular community – prokaryotes
others
