# Supplementary material for: Metagenomic analysis provides functional insights into seasonal change of a non-cyanobacterial prokaryotic community in temperate coastal waters
Source: PLoS One. 2021 Oct 12;16(10):e0257862. doi: 10.1371/journal.pone.0257862 (PMC8509957; doi:10.1371/journal.pone.0257862)
Supplement: S1 Table — The values in brackets indicate the percentage relative to the total number of sequences of prokaryotes other than cyanobacteria. (PDF) [file pone.0257862.s008.pdf]

year	month	site	raw read	QC-passed read	predicted peptide sequence				
					total	assigned to domain	sequences of prokaryotes other than cyanobacteria		
							total	assigned to KEGG Orthology	assigned to KEGG pathway
2012	Apr	C5	1066455	653884	379568	195139	188436	99610 (52.9%)	71142 (37.8%)
		C12	976768	602691	339951	164291	157835	83278 (52.8%)	59204 (37.5%)
	May	C5	1130907	744463	412845	186033	174551	93121 (53.3%)	66490 (38.1%)
		C12	1026386	703451	405772	177567	164650	86029 (52.2%)	61008 (37.1%)
	Jun	C5	2043365	1407262	728475	293543	156267	74149 (47.5%)	53194 (34.0%)
		C12	1479041	1064798	592436	242070	133166	67138 (50.4%)	48726 (36.6%)
	Jul	C5	1546471	1112637	664212	288955	236106	128305 (54.3%)	93178 (39.5%)
		C12	1671821	1198896	679884	248094	169258	86330 (51.0%)	63127 (37.3%)
	Aug	C5	1754438	1187968	739189	392418	324943	180767 (55.6%)	129791 (39.9%)
		C12	1769189	1299866	809971	420931	198820	90474 (45.5%)	65642 (33.0%)
	Sep	C5	1498167	1021261	577434	266950	140693	76704 (54.5%)	55977 (39.8%)
		C12	1527646	1076348	656375	338288	174778	91178 (52.2%)	65876 (37.7%)
	Nov	C5	1615842	1126761	619814	234052	113786	60206 (52.9%)	43559 (38.3%)
		C12	1582212	1111109	573600	191560	97420	51149 (52.5%)	37545 (38.5%)
2013	Jan	C5	1431831	1042610	582243	248818	208132	116827 (56.1%)	85199 (40.9%)
		C12	1468742	1044928	501228	160477	101001	55169 (54.6%)	40011 (39.6%)
	Mar	C5	2164583	1375860	820829	381390	352959	193966 (55.0%)	138673 (39.3%)
		C12	2468767	1761064	1022587	438672	397849	222019 (55.8%)	160907 (40.4%)
	Apr	C5	2470493	879973	528281	238137	219370	118988 (54.2%)	84668 (38.6%)
		C12	1434920	967605	583654	311669	294757	161462 (54.8%)	71142 (37.8%)
	Jun	C5	1985865	1457783	850292	391236	201876	104979 (52.0%)	59204 (37.5%)
		C12	1478470	939608	556490	260664	163956	86573 (52.8%)	66490 (38.1%)
