# Supplementary material for: Metagenomic analysis provides functional insights into seasonal change of a non-cyanobacterial prokaryotic community in temperate coastal waters
Source: PLoS One. 2021 Oct 12;16(10):e0257862. doi: 10.1371/journal.pone.0257862 (PMC8509957; doi:10.1371/journal.pone.0257862)
Supplement: S1 Fig — The labels indicate “broad category → KEGG category → KEGG pathway”. Only KEGG pathways with max abundance > 0.02 are shown. As some genes are assigned to more than one pathway, the total is greater than 1.0. (PDF) [file pone.0257862.s001.pdf]

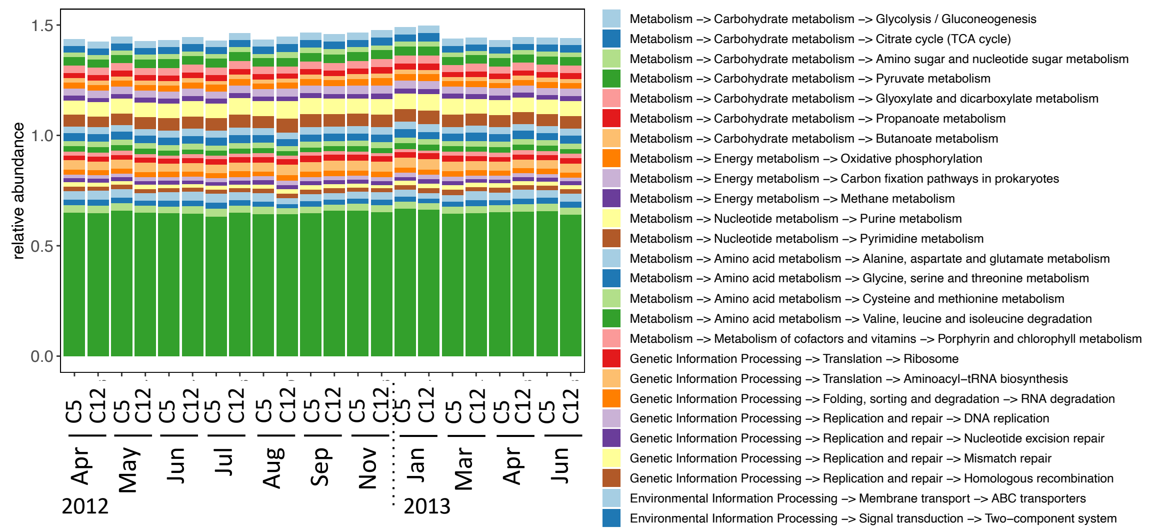

relative abundance
0.0
0.5
1.0
1.5
C5
C12
Apr
May
Jun
Jul
Aug
Sep
Nov
Jan
Mar
2012
2013
Metabolism –> Carbohydrate metabolism –> Glycolysis / Gluconeogenesis
Metabolism –> Carbohydrate metabolism –> Citrate cycle (TCA cycle)
Metabolism –> Carbohydrate metabolism –> Amino sugar and nucleotide sugar metabolism
Metabolism –> Carbohydrate metabolism –> Pyruvate metabolism
Metabolism –> Carbohydrate metabolism –> Glyoxylate and dicarboxylate metabolism
Metabolism –> Carbohydrate metabolism –> Propanoate metabolism
Metabolism –> Carbohydrate metabolism –> Butanoate metabolism
Metabolism –> Energy metabolism –> Oxidative phosphorylation
Metabolism –> Energy metabolism –> Carbon fixation pathways in prokaryotes
Metabolism –> Energy metabolism –> Methane metabolism
Metabolism –> Nucleotide metabolism –> Purine metabolism
Metabolism –> Nucleotide metabolism –> Pyrimidine metabolism
Metabolism –> Amino acid metabolism –> Alanine, aspartate and glutamate metabolism
Metabolism –> Amino acid metabolism –> Glycine, serine and threonine metabolism
Metabolism –> Amino acid metabolism –> Cysteine and methionine metabolism
Metabolism –> Amino acid metabolism –> Valine, leucine and isoleucine degradation
Metabolism –> Metabolism of cofactors and vitamins –> Porphyrin and chlorophyll metabolism
Genetic Information Processing –> Translation –> Ribosome
Genetic Information Processing –> Translation –> Aminoacyl−tRNA biosynthesis
Genetic Information Processing –> Folding, sorting and degradation –> RNA degradation
Genetic Information Processing –> Replication and repair –> DNA replication
Genetic Information Processing –> Replication and repair –> Nucleotide excision repair
Genetic Information Processing –> Replication and repair –> Mismatch repair
Genetic Information Processing –> Replication and repair –> Homologous recombination
Environmental Information Processing –> Membrane transport –> ABC transporters
Environmental Information Processing –> Signal transduction –> Two−component system
Cellular Processes –> Cellular community − prokaryotes –> Quorum sensing
others
