# Supplementary material for: Metagenomic analysis provides functional insights into seasonal change of a non-cyanobacterial prokaryotic community in temperate coastal waters
Source: PLoS One. 2021 Oct 12;16(10):e0257862. doi: 10.1371/journal.pone.0257862 (PMC8509957; doi:10.1371/journal.pone.0257862)
Supplement: S6 Fig — The KEGG categories are arranged in the same order as in S4 Fig. (PDF) [file pone.0257862.s006.pdf]

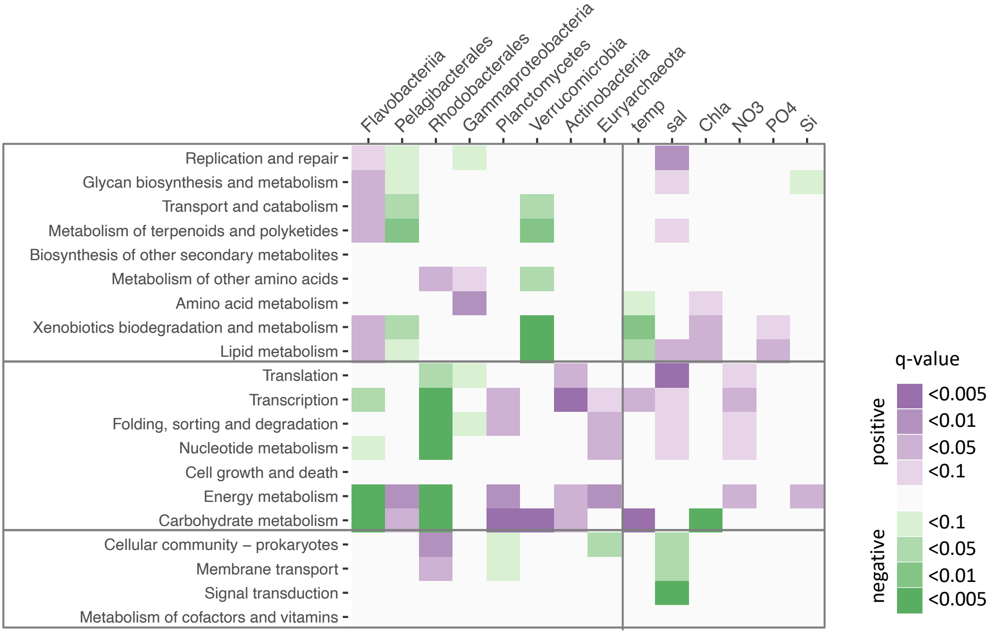
Flavobacteriia
Pelagibacterales
Rhodobacterales
Gammaproteobacteria
Planctomycetes
Verrucomicrobia
Actinobacteria
Euryarchaeota
temp
sal
Chla
NO3
PO4
Si
Replication and repair
Glycan biosynthesis and metabolism
Transport and catabolism
Metabolism of terpenoids and polyketides
Biosynthesis of other secondary metabolites
Metabolism of other amino acids
Amino acid metabolism
Xenobiotics biodegradation and metabolism
Lipid metabolism
Translation
Transcription
Folding, sorting and degradation
Nucleotide metabolism
Cell growth and death
Energy metabolism
Carbohydrate metabolism
Cellular community – prokaryotes
Membrane transport
Signal transduction
Metabolism of cofactors and vitamins
q-value
positive
<0.005
<0.01
<0.05
<0.1
negative
<0.1
<0.05
<0.01
<0.005
